# Unrecognized Myocardial Infarction Assessed by Cardiac Magnetic Resonance Imaging – Prognostic Implications

**DOI:** 10.1371/journal.pone.0148803

**Published:** 2016-02-17

**Authors:** Anna M. Nordenskjöld, Per Hammar, Håkan Ahlström, Tomas Bjerner, Olov Duvernoy, Kai M. Eggers, Ole Fröbert, Nermin Hadziosmanovic, Bertil Lindahl

**Affiliations:** 1 Department of Cardiology, Faculty of Health, Örebro University, Örebro, Sweden; 2 Department of Radiology, Västmanland Hospital Västerås, Västerås, Sweden; 3 Department of Radiology, Oncology and Radiation Science, Uppsala University, Uppsala, Sweden; 4 Department of Medical Sciences, Cardiology, Uppsala University, Uppsala, Sweden; 5 Uppsala Clinical Research Centre, Uppsala, Sweden; Medstar Washington Hospital Center, UNITED STATES

## Abstract

**Background:**

Clinically unrecognized myocardial infarctions (UMI) are not uncommon and may be associated with adverse outcome. The aims of this study were to determine the prognostic implication of UMI in patients with stable suspected coronary artery disease (CAD) and to investigate the associations of UMI with the presence of CAD.

**Methods and Findings:**

In total 235 patients late gadolinium enhancement cardiovascular magnetic resonance (LGE-CMR) imaging and coronary angiography were performed. For each patient with UMI, the stenosis grade of the coronary branch supplying the infarcted area was determined. UMIs were present in 25% of the patients and 67% of the UMIs were located in an area supplied by a coronary artery with a stenosis grade ≥70%. In an age- and gender-adjusted model, UMI independently predicted the primary endpoint (composite of death, myocardial infarction, resuscitated cardiac arrest, hospitalization for unstable angina pectoris or heart failure within 2 years of follow-up) with an odds ratio of 2.9; 95% confidence interval 1.1–7.9. However, this association was abrogated after adjustment for age and presence of significant coronary disease. There was no difference in the primary endpoint rates between UMI patients with or without a significant stenosis in the corresponding coronary artery.

**Conclusions:**

The presence of UMI was associated with a threefold increased risk of adverse events during follow up. However, the difference was no longer statistically significant after adjustments for age and severity of CAD. Thus, the results do not support that patients with suspicion of CAD should be routinely investigated by LGE-CMR for UMI. However, coronary angiography should be considered in patients with UMI detected by LGE-CMR.

**Trial Registration:**

ClinicalTrials.gov NTC01257282

## Introduction

A myocardial infarction (MI) may be asymptomatic or associated with atypical symptoms unrecognized by the patient or by health professionals as indicative of MI. Such clinically unrecognized myocardial infarctions (UMIs) are not uncommon [1,2]. The presence of pathological Q-waves in the electrocardiogram (ECG) as indicators of UMI has been investigated in large population based studies. The reported prevalence of ECG detected UMIs has varied depending on the cohorts studied. In individuals 45–93 years of age, the reported prevalence is between 5–44% [1–3] and in patients with stable coronary artery disease (CAD) between 8–36% [4–6]. Patients with ECG detected UMI have a similar long-term prognosis as patients with clinically recognized MIs [1].

In recent years, late gadolinium enhancement cardiovascular magnetic resonance (LGE-CMR) imaging has facilitated detection of very small scars due to MI [7]. LGE-CMR is more sensitive for the detection of UMI than an ECG [3,5,6,8,9], conventional echocardiography [10] or nuclear scintigraphic techniques [11]. In studies of community dwellers without previously known MI, the prevalence of LGE-CMR detected UMI has been 6–30% [3,9,12,13]. In patients with suspected CAD, we and others have shown a prevalence of LGE-CMR detected UMI ranging from 19–27% [5,6,14]. Furthermore, we have recently shown that the majority of the LGE-CMR detected UMIs are located in a myocardial segment supplied by a coronary artery with a significant stenosis [14].

The prognostic implication of LGE-CMR detected UMIs in patients with suspected CAD has been evaluated previously in two small studies, in which UMI was associated with surprisingly high risks of future morbidity and mortality [5,6].

In this study our purpose was twofold; firstly, to examine the prognostic implication of LGE-CMR detected UMI in patients with stable suspected CAD without previously diagnosed MI; and secondly, to investigate the relation between UMI and future events to the extent, severity and localization of CAD.

## Methods

### Study Population

The details of the prospective multicentre study, Prevalence and prognostic value of Unrecognised Myocardial Injury in stable coronary artery disease (PUMI) have been previously reported [14–16]. Briefly, 265 patients with stable suspected CAD scheduled for elective coronary angiography were prospectively enrolled. Exclusion criteria were: pathological Q-wave in a 12-lead ECG, previously known MI, previous percutaneous coronary intervention (PCI) or coronary artery bypass grafting (CABG), history of congestive heart failure, estimated glomerular filtration rate below 30 ml/min/1.73 m^2^ or conditions contraindicating CMR or lack of suitability for participation in the study for any reason judged by the investigator. Patients were enrolled at six Swedish hospitals from January 2008 to March 2011. All patients provided written informed consent.

The calculation of the study sample size anticipated a 25% prevalence of UMI in patients with stable CAD [9], a 5-fold increased risk regarding the primary endpoint compared to subjects without UMI [5] and 2-year risk for the primary endpoint of 6% according to data from the Swedish Coronary Angiography and Angioplasty Registry [17]. The two first assumptions imply an event rate of 15% and 3% in the UMI and non UMI group, respectively. To show a statistically significant difference at the 5% level (two-sided test) with 80% power, 61 and 183 patients (total = 243 patients) with and without UMI, respectively, were needed.

The study was approved by the Ethical Review Board in Uppsala, Sweden (Dnr 2007/214) and conformed to the principles of the declaration of Helsinki. The PUMI study is registered at ClinicalTrials.gov (NCT01257282).

### Study Procedure

**Fig 1:**
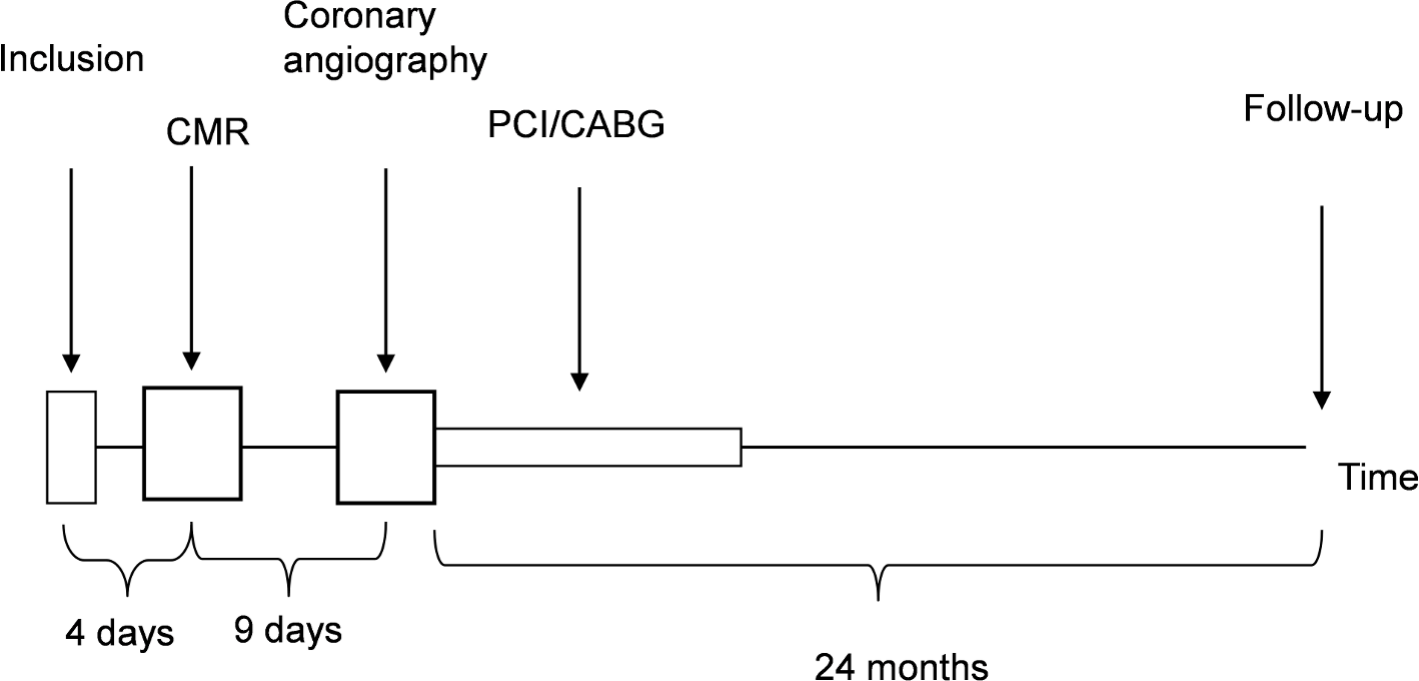
Flow chart for the patients in the PUMI study.

After study inclusion each patient´s clinical history was obtained, a physical examination performed and blood-samples drawn. At a median of four days after enrolment (inter quartile range (IQR) 0–11 days) LGE-CMR imaging was performed. Coronary angiography was performed at a median of nine days after the LGE-CMR (IQR 7–15 days). Medical treatment and revascularization was at the discretion of the physicians in charge. All patients were followed up by telephone interview, review of hospital records and/or death certificates 24–26 months after inclusion (Fig 1). Data regarding MI, death, resuscitated cardiac arrest, subsequent revascularization, hospitalisations for unstable angina pectoris, congestive heart failure and other heart diseases were recorded and events were registered as first occurring. Our pre-specified primary endpoint was a composite of death, resuscitated cardiac arrest, MI and hospitalisation for congestive heart failure or unstable angina at 2 years. Out of the 265 patients included in the study, 235 had a coronary angiography and a CMR investigation of technically adequate quality to enable analysis. These 235 patients form the basis for the present study. Reasons for drop-out have been described previously [14].

### Electrocardiogram

A 12-lead resting ECG was obtained at inclusion. ECG changes were classified according to the Minnesota Code Classification System for Electrocardiographic Findings [18].

### Cardiac Magnetic Resonance and Coronary Angiography

Acquisition and analysis of CMR image investigations and coronary angiographies have previously been described in detail [14]. Briefly, clinical 1.5-T scanners (Philips Intera, Best, the Netherlands; Philips Achieva, Best, the Netherlands or Siemens Symphony, Erlangen, Germany) were used to perform CMR-examinations with a general scanning protocol consisting of cine short axis images and a viability sequence in short axis, long axis 2-chamber, 3-chamber and 4-chamber views using ECG-triggering and breath-holding. Viability imaging was performed with a minimum delay of 15 minutes after intravenous administration of 0.15 ml/kg bodyweight (maximum dose 15 ml) of gadobutrol (Gadovist®, Bayer, Leverkusen, Germany). The viability sequence was a 3D inversion recovery gradient echo sequence with the following parameters: repetition time set to shortest (typically 4.0–4.2 ms), echo time set to shortest (typically 1.18–1.28 ms), inversion time chosen by the operator to null normal myocardium, flip angle 15°, image matrix 256x100, field-of-view 375 x 281 mm, reconstructed voxel size 0.73x0.73x5 mm. Eleven slices were acquired per breath hold for the long axis slices and 22 slices divided in two breath holds for the short axis. Each breath hold was 16 seconds at heart rate 60 bpm.

The myocardium was divided into 17 segments according to the American Heart Association model proposed by Cerqueira et al [19]. Two radiologists (T.B and P.H) individually reviewed the CMR Images, unaware of the patients´ clinical history, for localized areas of late gadolinium enhancement (LGE) visible in at least two imaging planes. LGE with a subendocardial component indicated UMI, while in patients without LGE or a LGE area without a subendocardial component an UMI was not diagnosed. In case of disagreement between the two reviewers a final decision was made in consensus. The physician in charge of the patient had no access to the results of the analysis of the CMR with the exception of left ventricle ejection fraction (LVEF) and wall motion abnormalities.

Coronary angiography was performed with standard projections. The coronary angiograms were analyzed by two radiologists (O.D and P.H), blinded to the patients’ clinical history and to the results of LGE-CMR. The coronary vessels were divided into 19 segments, derived from the 16 segment model proposed by Austen [20]. The degree of diameter narrowing in each of the 19 coronary segments was visually categorized as 0–29%, 30–49%, 50–69%, 70–99% or 100% (occlusion). In case of disagreement between the two reviewers a final decision was made in consensus. If a coronary artery had a stenosis of ≥30% we visually assessed, by taking the individual coronary anatomy in consideration, which of the myocardial segments in the 17-segment model by Cerqueira et al [19] that were supplied by the artery downstream of the lesion. A stenosis with ≥70% narrowing was considered hemodynamically significant. An association between coronary artery stenosis and LGE was therefore registered when the myocardial segment(s) with LGE were supplied by a coronary artery with a ≥70% stenosis.

The number of vessels affected by a ≥70% stenosis also determined the extent of atherosclerosis. No systematical assessments of fractional flow reserve (FFR) were made.

### Statistical Analyses

All analyses were pre-defined in the primary objective for the present study. No post-hoc analyses were made. The continuous data were not normally distributed and are presented as median and IQR. Mann Whitney U-test was used. Comparisons between categorical data were made using either Chi-square tests or Fisher´s exact test. Age was analysed both as a continuous variable and as a categorical variable. UMI, gender, degree of CAD, extent of CAD and matched UMI were analysed as categorical variables. In order to identify the clinical characteristics associated with UMI and the relationship with the primary endpoint, univariable and multivariable logistic regression analyses were performed. Given the limited number of events, four different models with a maximum of three covariates in each model were created. All models were adjusted for age. Model 1 also included sex while models 2 to 4 included coronary stenosis >70%, extent of CAD and matched UMI, respectively. Results are presented as odds ratios (OR) with 95% confidence intervals (CIs). All statistical tests were two-tailed and p<0.05 was regarded as statistically significant.

Data analyses were performed using the SAS (version 9.4; SAS Institute, Cary, North Carolina) or the Predictive Analytical SoftWare (PASW statistics 17.03) program (SPSS Inc, Chicago, IL, USA).

## Results

### Clinical Characteristics, Coronary Artery Disease and Prevalence of Unrecognized Myocardial Infarction

UMIs were found in 58 patients (25%). The clinical characteristics of the study population and the findings at the coronary angiography stratified according to the presence of UMIs are shown in Table 1. The median age of the entire group was 65.4 years (IQR 59.9–70.6 years), 34% were women and 52% had symptoms indicating stable CAD for more than 12 months. The median LVEF was 66% (IQR 62–72%).

**Table 1:** Clinical characteristics.

Characteristic	All patients	No UMI	UMI	p-value
**Number**	235	177	58	
**Age** in years, median (IQR)	65 (60–71)	65 (60–70)	66 (64–72)	0.06
**Women** (%)	80 (34)	66	14	0.08
**CAD risk factors**				
Waist circumference, cm, median (IQR)	100 (93–107)	99 (92–106)	103 (95–109)	0.04
BMI (kg/m2), median (IQR)	27 (25–30)	27 (25–29)	28 (25–30)	0.29
Family history of CAD (%)	117 (50)	87	30	0.76
Previous or present smoking (%)	143 (61)	105	38	0.40
Hypertension (%)	132 (56)	94	38	0.13
Diabetes mellitus (%)	49 (21)	32	17	0.09
**Symptoms of angina pectoris**				
Less than 2 months (%)	7 (3)	6	1	0.85
2–12 months (%)	105 (45)	80	25	
More than 12 months (%)	123 (52)	91	32	
CCS class 0 (%)	9 (4)	5	4	0.59
CCS class 1 (%)	70 (30)	51	19	
CCS class 2 (%)	110 (47)	85	25	
CCS class 3 (%)	45 (19)	35	10	
**Medications**				
Aspirin (%)	211 (90)	158	53	0.81
Clopidigrel (%)	7 (3)	4	3	0.37
Warfarin (%)	7 (3)	6	1	1.00
Beta blocker (%)	161 (69)	117	44	0.19
ACE-I or AT-II (%)	84 (36)	58	26	0.12
Calcium channel blocker (%)	56 (24)	37	19	0.08
Long-acting nitrate (%)	59 (25)	37	22	**0.01**
Statin/other lipid lowering agent (%)	167 (71)	121	46	0.13
**Cardiac magnetic resonans imaging**				
Ejection fraction, median (IQR)	66 (62–72)	66 (61–71)	67 (62–72)	0.43
**Coronary angiography**				
Stenosis >70% (%)	135 (57)	88	47	**<0.001**
Three vessel disease (%)	23 (10)	9	14	**<0.001**
Stenosis <50% (%)	92(39)	82	10	**<0.001**
**Revascularized after angiography**				
PCI (%)	98 (42)	61	37	**<0.001**
CABG (%)	23 (10)	15	8	0.42

ACE-I = Angiotensin-Converting-Enzyme Inhibitor

AT-II = Angiotensin II receptor antagonist; BMI = Body Mass Index

CABG = Coronary Artery Bypass Grafting; CAD = Coronary Artery Disease

CCS = Canadian Cardiovascular Society; PCI = Percutaneous Coronary intervention

UMI = Unrecognized Myocardial Infarction.

As previously reported [14] the UMIs were predominately located in the inferior and inferior-lateral myocardial segments (AHA segments 4, 5,10,11,15,16) with 56% of UMIs located in those areas. Coronary angiography documented at least one significant stenosis in 57% of the patients and 10% of the patients had three vessel disease. Patients with UMIs more often had one or more significant coronary artery stenosis than patients without UMI, 81% vs. 50% (p<0.001). Patients with UMI, compared to patients without UMI, were more likely to have three vessel disease, 24% vs. 5% (p<0.001). In 67% (39/58) of the patients with UMI, the UMI was downstream of a significant stenos/occlusion and in 33% (19/58) the UMI had no relation to a significant stenosis. The proportion of UMI downstream a ≥70% stenosis tended to be unevenly distributed between RCA, LCX and LAD, 25.0%, 18.7% and 10.8%, respectively (p = 0.08).

After the coronary angiography, 121 patients (52%) underwent coronary artery revascularization; 42% with PCI and 10% with CABG.

### UMI and Prognosis

During the follow-up period of 24–26 months (median 754 days, IQR 736–790), 18 patients reached the primary composite endpoint (7.7%), five patients died (2.1%), four developed acute MIs (1.7%), seven had episodes of unstable angina pectoris (3.0%) and two were hospitalised for heart failure (0.9%). One patient experienced a second event during follow-up (not included as an event in the primary endpoint); an acute MI 21 months after an episode of unstable angina pectoris. No patient was lost to follow-up.

**Fig 2:**
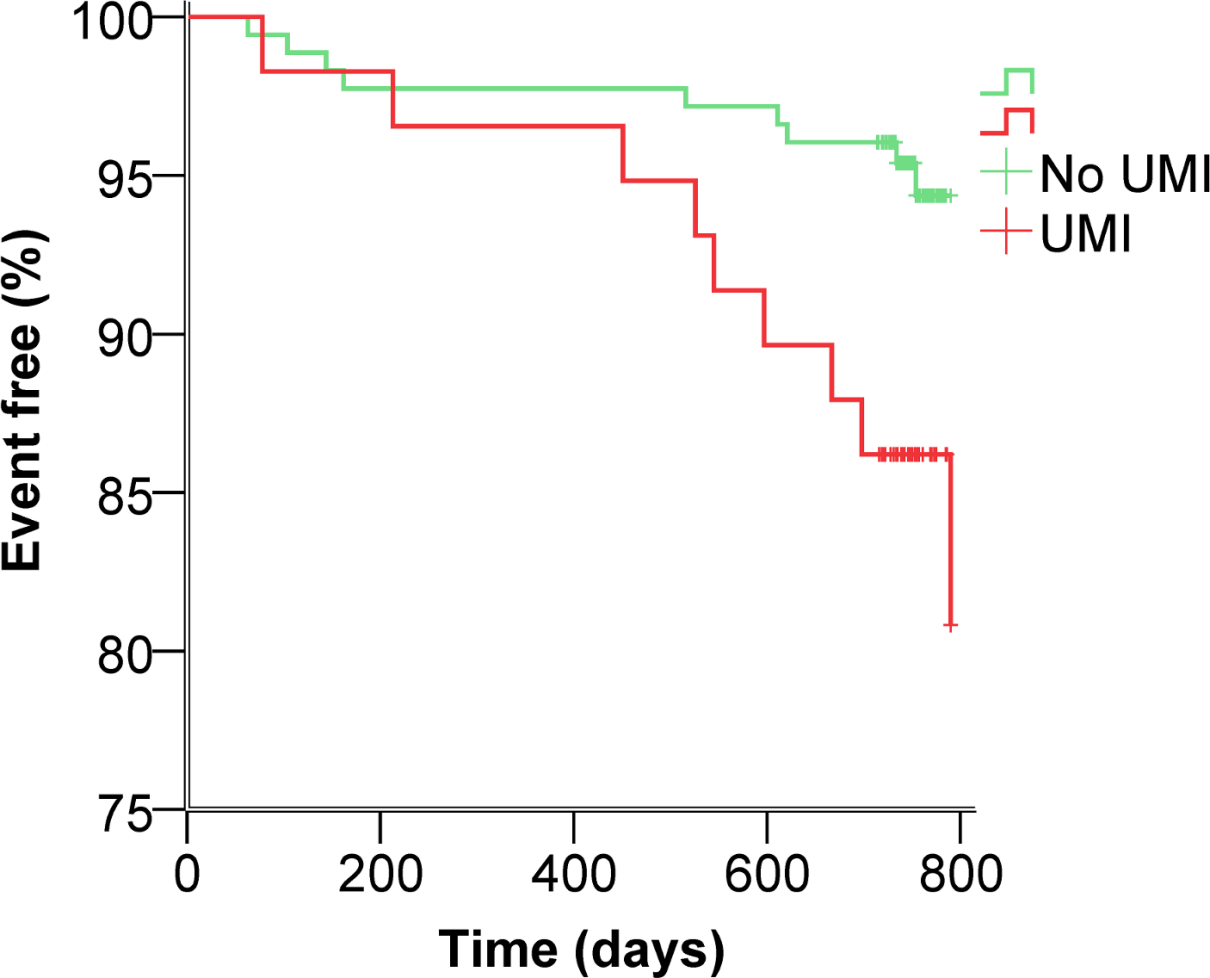
Kaplan-Meier plot of the cumulative probability of remaining event free for patients with UMI versus patients without UMI. P = 0.014. Green line = no UMI. Red line = UMI.

Of patients with UMI, 15.5% (9/58) reached the primary endpoint compared to 5.1% (9/177) of patients with no UMI (OR 3.4, 95% CI 1.3–9.1, p = 0.014) (Table 2 and Fig 2).

**Table 2:** Proportion of patients reaching the primary composite endpoint in relation to patient characteristics.

Characteristic		Endpoint	P-value
**Age**	< Median age 65.4 years (no = 117)	6.0%	p = 0.34
** **	≥ Median age 65.4 years (no = 118)	9.3%	
**Gender**	Men (no = 155)	9.7%	p = 0.12
** **	Women (no = 80)	3.8%	
**CMR image**	With UMI (no = 58)	15.5%	**p = 0.014**
** **	Without UMI (no = 177)	5.1%	
**Coronary angiography**	Significant coronary stenosis (no = 135)	11.9%	**p = 0.013**
** **	No significant coronary stenosis (no = 100)	2.0%	
** **	Extent of coronary artery disease		
** **	No ≥70% stenosis (no = 100)	2.0%	
** **	One vessel disease (no = 67)	4.5%	p = 0.37*
** **	Two vessel disease (no = 45)	20.0%	**p = 0.002**
** **	Three vessel disease (no = 23)	17.4%	**p = 0.010**
**UMI and stenosis**	UMI with matched stenosis (no = 39)	15.4%	p = 0.97
	UMI and no matched stenosis (no = 19)	15.8%	

* In comparison with patients with no ≥70% stenosis.

Patients with an anatomic match between UMI and coronary artery stenosis reached the primary endpoint in 15.4% (6/39) compared to 15.8% (3/19) in patients with UMI without a match (p = 0.97). The five patients’ deaths resulted in an overall average annual mortality rate of 1.1% per year. Among patients with UMI, there was 1 death, average mortality 0.9% per year.

Besides UMI, the following factors were statistically significant univariate predictors of prognosis (Table 2): the presence of a significant coronary artery stenosis (OR 6.6, 95% CI 1.5–29.4, p = 0.013), the extent of CAD (one vessel disease OR 2.3, 95% CI 0.4–14.1, p = 0.37; two vessel disease OR 12.3, 95% CI 2.5–59.4, p = 0.002; and three vessel disease OR 10.3, 95% CI 1.8–60.4, p = 0.010).

The prognostic value of UMI was assessed by multivariable logistic regression in different models adjusting for clinical and angiographic factors (Table 3). UMI was an independent predictor for the primary endpoint after adjustment for age and gender. However, after adjustment for age and the presence of a significant coronary stenosis or extent of CAD, UMI was no longer statistically significantly associated with outcome.

**Table 3:** Multivariable logistic regression analyses of the primary endpoint as outcome variable. Model 1: unrecognized myocardial infarction (UMI), age and gender. Model 2: UMI, age and ≥70% stenosis. Model 3: UMI, age and the extent of coronary artery disease (CAD). Model 4: age, UMI in an area supplied by a coronary artery with a ≥70% stenosis (UMI with match) and UMI in an area supplied by a coronary artery without a ≥70% stenosis (UMI without match). Presented as OR and 95% CI.

Covariates	Model 1	Model 2	Model 3	Model 4
**UMI**	2.92	2.42	2.12	-
** **	1.08–7.89	0.88–6.61	0.73–6.13	
**Age**	1.06	1.04	1.04	1.06
** **	0.99–1.13	0.98–1.11	0.97–1.11	0.99–1.13
**Female gender**	0.40	-	-	-
** **	0.11–1.45			
**≥70% stenosis**		4.46	-	-
** **		0.96–20.7		
**Extent of CAD**				
No ≥70 % stenosis	-	-	reference	-
One-vessel disease	-	-	1.76	-
			0.28–11.1	
Two-vessel disease	-	-	8.72	-
			1.72–44.2	
Three-vessel disease	-	-	5.89	-
** **			0.91–38.2	
**Matched UMI**				
No UMI				reference
UMI with match	-	-	-	3.13
				1.04–9.48
UMI without match	-		-	3.06
** **				0.73–12.8

OR; Odds ratio, CI; confidence interval.

## Discussion

The main findings of the present study were twofold. Firstly, the presence of UMI was associated with a significant threefold risk of adverse events during follow-up in univariate analysis. However, after adjustment for age and severity of CAD or extent of CAD, the increased risk associated with UMI did not remain statistically significant. Secondly, no significant difference in event rate was noted for patients with UMI with or without a significant stenosis in the coronary artery supplying the myocardial segment affected.

To the best of our knowledge, this is the first prospective study evaluating the prognostic implication of LGE-CMR detected UMI, with or without a significant stenosis in the coronary artery supplying the affected myocardial segment. We hypothesised that an UMI located in an area supplied by a ≥70% stenosed coronary artery would be associated with worse prognosis and indicate a more aggressive disease. However, at odds with our hypothesis there was no difference in event rates between patients with UMIs with or without a significant stenosis in the coronary artery supplying the affected myocardial segment.

The pathophysiological mechanisms causing UMIs are not completely understood, but there is a statistically significant association between UMI and the extent and severity of CAD [6,14]. The progression of atherosclerosis may include repeated silent plaque ruptures and thrombosis [21,22], which might occasionally cause UMI, followed by wound healing, with an increase in plaque burden and narrowing of stenoses [23]. Thus, the presence of UMI may indicate the progression of atherosclerosis into more severe and widespread CAD. The absence of a statistically significant difference in event rates between UMIs with or without a significant stenosis in the coronary artery supplying the affected myocardial segment in the present study may be due to our small number of events or as plaques (obstructive or non-obstructive) generating new events may be located anywhere in the coronary tree. Most future MIs develop from mild to moderate stenoses [24,25] and the total coronary stenosis burden has been shown to predict the incidence of subsequent cardiac events better than the number of high-grade stenoses [26]. Furthermore, severe stenoses are more often associated with protective collateral circulation [24]. The occurrence of UMIs in individuals without significant CAD is more difficult to explain, but possible mechanisms include: coronary artery spasm [27], coronary embolism [28] and type 2 MI [29].

These data relate to the evidence from previous studies on UMI in patients with suspected CAD and differences in results. Importantly, the average annual mortality rate in our study was approximately 1% in patients with and without UMI, which is in accordance with estimates of the annual mortality of 1.2–2.4% in patients with stable CAD [30] derived from global clinical trials of anti-anginal and preventive therapy. A recent study based on patients with stable CAD in two different cohorts with 4.8 and 6.6 years of follow-up, demonstrated an annual risk for cardiovascular events (MI, stroke or cardiovascular death) of 2.2 and 3.4%, respectively [31].

In two previous studies of the prognostic impact of LGE-CMR detected UMI, the mortality in the UMI-groups were approximately 11% [6] and 22% per year [5] as estimated from the hazard ratios reported. This discrepancy in mortality between previous studies and ours may be due to differences in patient characteristics. In the study by Kwong et al. [5], all patients were referred to CMR on clinical grounds and patients with a clinically motivated CMR may differ from those undergoing a standard evaluation for suspected CAD, and may include more of those with unusual clinical presentations and/or multiple cardiac issues. In contrast to our study, the cohort of Kwong et al. [5] also included patients who had undergone coronary revascularization with PCI and/or CABG before CMR. The revascularization procedures might have resulted in procedure related myocardial injuries registered as UMI resulting in a too high UMI prevalence [32]. In the study by Kim et al. [6], the selection of patients was more similar to that in the present study. No patients had previous coronary interventions and the CMR was performed for the purpose of research only. Despite this, there were considerable differences in clinical characteristics with higher prevalence’s of CAD risk factors and medical treatment at baseline between the patients in the study by Kim et al.[6] and our patients. Furthermore, a large proportion of our UMI patients received revascularization shortly after the coronary angiography, whereas a lower proportion of the UMI patients in the study by Kim et al [6] underwent revascularization sometime during the follow-up period, which might explain the differences in mortality rates. However, the effect of revascularisation on the survival of patients with stable CAD is debated [33].

In agreement with the present study, Kim et al. [6] demonstrated an association between UMI and extent and severity CAD in univariate analysis. But unlike the present study they did not adjust the data concerning UMI and prognosis for the extent and severity of CAD, neither did they investigate the relationship between the localization of significant coronary artery stenoses and the localizations of UMIs.

### Clinical Implications

Clearly, prognosis is mainly influenced by the extent and severity of CAD but we cannot exclude that the presence of UMI even without significant CAD may infer independent prognostic information. However, in contrast to CAD, we lack established treatment to decrease the risk for new cardiac events associated with the presence of UMI. Therefore, our results do not support that patients with suspicion of CAD should be routinely investigated by LGE-CMR for UMI. However, the results do have a clinical application when it comes to en passant detected UMIs in patients undergoing cardiac LGE-CMR. Given the association with prognosis and the close relationship between UMI and significant CAD, a coronary angiography should be considered if the patients have cardiac symptoms.

Additional, larger clinical studies are needed to determine the prognostic implication of UMI in patients without CAD.

### Limitations

Although the present study included more patients than previous studies [5,6], the number of events was low giving limited power to show significant associations. The low number of events prevented more complex multivariable models with simultaneous adjustment for several potentially relevant covariates.

This was a multicenter study with sites using different MRI scanners. The examination protocols were therefore not exactly the same for all examinations. Another limitation was that the assessment of coronary artery stenoses was done by visual grading of the lumen and that, FFR data on the fractional flow reserve were not available. Furthermore, the assessment of the myocardial segments affected by coronary artery stenoses was determined independently by two examiners taking into account the individual coronary anatomy. Unfortunately, there are no objective or universally accepted criteria for this analysis.

## Abbreviations

AHA: American Heart Association
CABG: coronary artery bypass grafting
CAD: coronary artery disease
CI: confidence interval
LGE-CMR: late gadolinium enhancement cardiovascular magnetic resonance imaging
ECG: electrocardiogram
FFR: fractional flow reserve
IQR: inter quartile range
LVEF: left ventricle ejection fraction
MI: myocardial infarction
OR: odds ratio
PCI: percutaneous coronary intervention
PUMI: prevalence and prognostic value of Unrecognised Myocardial Injury in stable coronary artery disease
SD: standard deviation
UMI: unrecognized myocardial infarction
